# Community midwives’ and health visitors’ experiences of research recruitment: a qualitative exploration using the Theoretical Domains Framework

**DOI:** 10.1017/S1463423621000050

**Published:** 2021-01-29

**Authors:** Jennie Rose, Kieran Lynn, Jane Akister, Fiona Maxton, Sarah A. Redsell

**Affiliations:** 1 Faculty of Health, Education, Medicine and Social Care, Anglia Ruskin University, Cambridge, UK; 2 Research Department, North West Anglia NHS Foundation Trust, Peterborough, UK; 3 School of Health Sciences, University of Nottingham, Nottingham, UK

**Keywords:** community midwives, health visitors, recruitment, Theoretical Domains Framework

## Abstract

**Background:**

Successful research is frequently hampered by poor study recruitment, especially in community settings and with participants who are women and their children. Health visitors (HVs) and community midwives (CMs) are well placed to invite young families, and pregnant and postnatal women to take part in such research, but little is known about how best to support these health professionals to do this effectively.

**Aim:**

This study uses the Theoretical Domains Framework (TDF) to explore the factors that influence whether HVs and CMs invite eligible patients to take part in research opportunities.

**Method:**

HVs (*n* = 39) and CMs (*n* = 22) working in four NHS Trusts and one community partnership in England completed an anonymous, online survey with open-ended questions about their experiences of asking eligible patients to take part in the research. Qualitative data were analysed using directed content analysis and inductive coding to identify specific barriers and enablers to patient recruitment within each of the 14 theoretical domains.

**Findings:**

Six key TDF domains accounted for 81% of all coded responses. These were (a) environmental context and resources; (b) beliefs about capabilities; (c) social/professional role and identity; (d) social influences; (e) goals; (f) knowledge. Key barriers to approaching patients to participate in the research were time and resource constraints, perceived role conflict, conflicting priorities, and particularly for HVs, negative social influences from patients and researchers. Enablers included feeling confident to approach patients, positive influence from peers, managers and researchers, beliefs in the relevance of this behaviour to health care and practice and good knowledge about the study procedures, its rationale and the research topic. The findings suggest that to improve research recruitment involving HVs and CMs, a package of interventions is needed to address the barriers and leverage the enablers to participant approach.

## Background

Healthcare professionals play an important role in the recruitment of participants in research studies. In the UK, data protection legislation prevents researchers from approaching potential participants directly (Redsell and Cheater, 2001; Preston *et al.*, 2016; Grady *et al.*, 2019), and therefore it is common practice for healthcare professionals to inform patients^1^ of research participation opportunities (Preston *et al.*, 2016). However, there is substantial evidence that when performing this function, healthcare professionals approach only a proportion of eligible patients (Bonevski *et al.*, 2014; Hughes-Morley *et al.*, 2015; Tromp and Vathorst, 2015; Briel *et al.*, 2016; Preston *et al.*, 2016). This introduces biases to the sample as well as adversely affecting recruitment (Preston *et al.*, 2016; Rose *et al.*, 2017).

Whilst there is a growing body of research into the factors that influence recruitment to research, there has been less of a focus on community healthcare settings, particularly where the participants are perinatal women and young children. (Frew *et al.*, 2014). Yet, research involving these participants can be especially susceptible to recruitment problems (Baxter *et al.*, 2012; Webster *et al.*, 2012; Jordan *et al.*, 2013; Pica and Bourgeois, 2016; Huntington *et al.*, 2017; van der Graaf *et al.*, 2018). Historically, women in their childbearing years were excluded from research participation, in case it was detrimental to their future children, and a prevailing precautionary approach may be a contributory factor (Frew *et al.*, 2014). Policies changed decades ago, but research involving these participants remains susceptible to low rates of accrual (Pica and Bourgeois, 2016; van der Graaf *et al.*, 2018) and undersampling of socio-economically disadvantaged and minority ethnic groups (Baxter *et al.*, 2012; Webster *et al.*, 2012; Jordan *et al.*, 2013; Huntington *et al.*, 2017). In the case of pregnant women, the narrow window of eligibility for recruitment presents a particular challenge (Coleman-Phox, 2013 #107). Other explanations also focus on issues that stem from the patients, such as lack of time (van Delft, 2013; Frew *et al.*, 2014) and competing priorities including childcare and work commitments (Daniels *et al.*, 2012; Carpenter, 2016). Much less attention has been paid to the healthcare professionals’ role in the recruitment process for this population (Tooher *et al.*, 2008). With early intervention to improve population health being high on the policy agenda in the UK and elsewhere, a greater understanding of the influence of healthcare professionals on the recruitment of perinatal women and children to research is needed.

In the UK, community midwives (CMs) and health visitors (HVs) (public health nurses) provide health care for women and their children, from pregnancy to 5 years of age. Delivering universal services, these practitioners have very high-potential reach (Laws *et al.*, 2016) and are well placed to approach pregnant women, new parents and families about participation in research. However, where HVs and CMs have been involved in participant recruitment, disappointing recruitment and limited representativeness of the study sample has been an issue resulting from reluctance of the healthcare professionals to approach all eligible participants (Hoddinott *et al.*, 2007; Knight and Wyatt, 2010; Mytton *et al.*, 2014; Redsell *et al.*, 2017). In order to address these problems, it is necessary to understand the particular issues that concern CMs and HVs when they are tasked with informing families in their care of opportunities to take part in the research. With the exception of one study, which looked at barriers to CMs identifying potential participants in a specific randomised controlled trial (RCT) (Stuart *et al.*, 2015), there is little previous research exploring the research recruitment experiences of these CMs and none that we could find focussing on the experiences of HVs.

The aim of this study was to explore HVs’ and CMs’ perceived barriers and enablers to approaching patients about research participation. We used an established theoretical framework, the Theoretical Domains Framework (TDF) (Michie *et al.*, 2005; Cane *et al.*, 2012; Michie *et al.*, 2014) to guide data collection and analysis. This evidence-based tool provides a systematic approach to understanding healthcare professionals’ behaviours and identifying what needs to change.

## Methods

We used the SRQR reporting guidelines (O’Brien *et al.*, 2014) to structure the reporting of this study.

### Design

We used a self-reported, anonymous, online, cross-sectional survey to collect data from the HV and CM participants. Eight questions gathered data about respondents’ professional and demographic characteristics. The remainder of the survey focussed on the specific behaviour of interest: approaching eligible patients about research participation. These questions were informed by the refined TDF (Cane *et al.*, 2012) (Table 1). An initial set of questions designed to elicit responses covering all 14 TDF domains was piloted with a convenience sample of healthcare professionals. Feedback from the pilot respondents prompted the rewording of some questions, and the addition of others, resulting in a broader set of open-ended questions, which sought to explore the possibility of barriers and facilitators that did not fit in any of the TDF domains as well as prompting the respondent to mention factors that would map to the theoretical domains (Supplementary file 1). The final questionnaire included 25 questions: 8 questions gathered demographic data and 17 questions invited free-text responses to questions about approaching patients about research participation. The redrafted survey was entered onto the host site (Jisc’s Online Surveys) and tested for functionality and comprehensibility by five health researchers employed in the authors’ Faculty, none of whom were part of the study team.

**Table 1.** The revised Theoretical Domain Framework and domain definitions

Domain	Definition^2^
Knowledge	An awareness of the existence of something
Skills	An ability or proficiency acquired through practice
Social/professional and role and identity	A coherent set of behaviours and displayed personal qualities of an individual in a social or work setting
Beliefs about capabilities	Acceptance of the truth, reality or validity about an ability, talent or facility that a person can put to constructive use
Beliefs about consequences	Acceptance of the truth, reality or validity about outcomes of a behaviour in a given situation
Reinforcement	Increasing the probability of a response by arranging a dependent relationship, or contingency, between the response and a given stimulus
Intentions	A conscious decision to perform a behaviour or a resolve to act in a certain way
Goals	Mental representations of outcomes or end state that an individual wants to achieve
Memory, Attention and Decision Processes	The ability to retain information, focus selectively on aspects of the environment and choose between two or more alternatives
Environmental Context and Resources	The ability to retain information, focus selectively on aspects of the environment and choose between two or more alternatives
Social influences	Interpersonal processes that can cause individuals to change their thoughts, feelings or behaviours
Optimism	The confidence that things will happen for the best or that desired goals will be attained
Emotion	A complex reaction pattern, involving experiential, behavioural and physiological elements, by which the individual attempts to deal with a personally significant matter or event
Behavioural regulation	Anything aimed at managing or changing objectively observed or measured actions

### Ethical approval

Permission to conduct the study was provided by the Anglia Ruskin University Faculty of Health, Social Care and Education Research Ethics Panel (Reference FHSCE_DREP-16-106) on 23 February 2017 and Health Research Authority approval (REC reference 17/HRA/1753) were granted on 10 April 2017. Local R&D permission was granted by four NHS Trusts and one social enterprise contracted to provide the NHS services.

### Participants and setting

We invited staff delivering community public health nursing (health visiting) and community midwifery services for four NHS Trusts and one social enterprise to complete the questionnaire. These organisations covered both rural and urban areas, in different regions of England. Prior experience of conducting research was required, however, the employing organisations did not have data on which staff had that experience, so all CMs and HVs were informed of the survey. Those who were eligible were identified through an initial filtering question on the survey.

### Researcher characteristics and reflexivity

At the time of data collection, the researchers included two registered nurses (SR and FM), one of whom is also a health visitor (SR), a social worker (JA) and a Research Fellow (JR). We all have experience working in non-academic roles with families, in clinical or community settings. We also all have experience of working with health and social care professionals to recruit participants to research projects, and these experiences prompted our interest in this study. Our application of an approach rooted in psychological theory to frame the study is influenced by our training as psychologists (JR, SR, KL).

### Data collection

Participating organisations sent an email to their HVs and CMs inviting them to take part in the study. A hyperlink in the email opened to the participant information sheet. Potential participants were informed that the survey was anonymous, no personally identifiable information would be captured and once submitted, their survey answers could not be withdrawn. Recipients were asked to confirm their consent before starting the survey and again before submitting their completed surveys. The survey was open for 4 weeks, and a reminder was sent after 2 weeks.

### Analysis

Data were downloaded from Jisc’s Online Surveys. Quantitative data were imported into SPSS Version 26 and analysed descriptively. Qualitative data were imported into NVivo Version 12 (QSR International Pty Ltd., 2018). Two researchers (JR and KL) independently coded text into each of the 14 theoretical domains of the refined TDF (Cane *et al.*, 2012). Responses were also examined for any barriers and enablers to approaching eligible patients about research participation that did not fit within any of the domains of TDF. The coders agreed on 99.1% of their coding decisions. A few differences in coding were discussed, and a consensus opinion was reached. Specific barriers and enablers to patient recruitment were then identified within each domain.

## Results

### Sample characteristics

A total of 22 CMs and 39 HV with experience of approaching patients about participation in research completed the survey. Employing organisations did not have data on the numbers of eligible staff, and it was therefore not possible to calculate a response rate. Participant characteristics are shown in Table 1. Most of the participants were females (*n* = 59, 97%) and 31 (52%) had more than 10 years of experience. Overall, the majority of participants were White British (*n* = 42, 69%) and working in urban environments (*n* = 39, 64%). Fifty percent of CM participants were Black, Asian and Minority Ethnic (BAME), as compared to 21% of HVs. Sixty-nine percent of HVs were working in economically deprived communities, as compared to 18% of CMs.

### Barriers and enablers to inviting eligible patients to take part in research

Across the dataset, 408 responses were mapped to the 14 TDF domains. Table 2 summarises the frequency of responses mapped to each of the 14 TDF domains for HV and CM participants. Across all 14 TDF domains, 21 barriers and 31 enablers were identified, plus 9 factors that could act as either a barrier or an enabler. We did not find any barriers or enablers that did not fit into one of the TDF domains (see Supplementary Material for the complete list of barriers and enablers identified in all 14 TDF domains).

**Table 2.** Characteristics of HV and CM participants

Participant characteristics	HV (*n* = 39)	CM (*n* = 22)	Total (*n* = 61)
Gender			
Female	37 (95%)	22 (100%)	59 (97%)
Male	1 (3%)	0 (0%)	1 (2%)
Prefer not to say	1 (3%)	0 (0%)	1 (2%)
Ethnicity			
BAME	8 (21%)	11 (50%)	19 (31%)
White	31 (79%)	11 (50%)	42 (69%)
Years of clinical experience			
Less than 10 years	21 (54%)	8 (46%)	29 (48%)
More than 10 years	18 (46%)	14 (64%)	32 (52%)
Description of current practice environment^1^			
Urban	28 (72%)	11 (50%)	39 (64%)
Rural	9 (23%)	7 (32%)	16 (26%)
Ethnically diverse	21(54%)	8 (36%)	29 (48%)
Economically deprived	27 (69%)	4 (18%)	31 (51%)
Affluent	7 (18%)	6 (27%)	13 (21%)

1 Participants could choose more than one option, so % does not add up to 100.

For both HVs and CMs, six key domains accounted for 81% of all coded responses. These were (a) environmental context and resources; (b) beliefs about capabilities; (c) social/professional role and identity; (d) social influences; (e) goals; (f) knowledge. The barriers and enablers for these key domains are detailed below with example quotations.

- Environmental context and resources

Across the dataset, environmental context and resources was the most frequently identified domain, and was apparent in the responses of 27 (69%) HVs and 18 CMs (81%). Specific barriers identified were heavy caseloads leaving insufficient time, insufficient staff, language barriers and challenging clinical situations. The most frequently cited barrier in this domain, evident in the responses of 48% of HVs and 61% of CMs, was heavy caseloads leaving insufficient time. Respondents felt they lacked the time to talk to patients about research opportunities. Staff shortages, leading to increased individual workloads, compounded the challenge of workload pressures, making it more difficult for HVs and CMs to find the time to talk to patients about research opportunities.

‘*It is difficult when the unit is busy and the time constraint, workload is high and staffing levels are poor*’ (community midwife)

‘*I find it difficult to find the time to enrol families for research due to busy workload*’ (health visitor)

HVs and CMs mentioned that language could also be a barrier when attempting to inform patients who spoke little or no English about potential research opportunities.

*‘It is a difficulty when English isn’t their first language*’ (health visitor)

The enabling effect of comprehensive and accessible study information was evident for both HVs and CMs; it was particularly important given the workloads and time constraints of these staff. They needed to feel equipped to answer the questions of patients about the research without having to find additional time in their schedules to read around the research topic.

‘*It’s fine as long as I have been given appropriate info myself in a concise form*’ (community midwife)

‘*A lot of the time due to time constraints and pressure from management we have little time to find out information so that we are able to answer questions that families may have*. (health visitor)

Some respondents suggested that if additional staff with specific responsibility for research were made available, more patients could be informed about research opportunities. Others felt that research funding should, but often didn’t, cover the financial cost of staff time needed to approach patients about research participation. These respondents felt that the cost was being borne by themselves, as it was added to their existing duties without the allocation of additional staff time to cover this work.

‘*Banging on again… TIME resource explicit and funded up front whether through bid process or combination of NHS Trust and monies from bid and CRN as required. But key weakness as appears Chief Investigators do not acknowledge the ‘cost’ of what NHS ‘jobbing’ clinical midwives need to be able to freely enjoy and support consistent good quality research recruitment*’ (community midwife)

*‘There should be payment to providers of care for payment of additional time for the research study recruitment’* (health visitor)

1. Social and professional role and identity

The second most frequently identified domain for both professional groups was professional role and identity. Across the dataset, there were different ways in which the respondents’ professional role and identity influenced their participant recruitment behaviour. There was an enabling belief expressed by both CMs and HVs that supporting research is integral to their professional role. However, some HVs felt strongly that it was not part of their professional role – this should be the researchers’ responsibility. A different subset of the HVs was somewhat ambivalent, suggesting that the research topic needed to relate to their role and practice, and noting there was potential for conflict with their professional role.

*‘I see it as a professional endeavour and one avenue into understanding the need of clients’* (health visitor)

*‘It is part of my job description’* (community midwife)

*‘Researchers should stop imposing on us and sort it out themselves’* (health visitor)

*‘I approve of encouraging participation in research as a general rule but am very respectful of the boundaries of roles, expectations and service policy’* (health visitor)

1. Social influences

Social influences could act as both barriers and enablers to the patient approach. This was more common for HVs than CMs, with 59% of HV participants compared to 45% of CMs having responses that mapped to this domain. The social influence of patients, which acted as a barrier, was much more frequently cited by HVs (*n* = 12, 31%) than by CMs (*n* = 2, 9%). These respondents actively chose whether to inform an eligible patient about a research study; it was a judgement based on the healthcare professionals’ perception of the patient’s situation rather than the implementation of the study’s eligibility criteria.

*‘I feel that you have to pick clients who you know would be willing to participate’* (health visitor)

*‘I wouldn’t ask them if I thought the client’s reaction might not be positive’* (community midwife)

Researchers who fail to engage with and support the health professionals were a barrier to the involvement of HVs, whereas CMs identified communicative and supportive researchers as an enabler. These healthcare professionals felt that it was the researchers’ responsibility not only to provide the information and physical resources for recruitment, but also to provide support and encouragement in person.

*‘We need more involvement from the researchers rather than just handing it to us!’* (health visitor)

*‘Researchers being visible and approachable, using easy to- understand language and making it relevant to our clinical area, and help in the recruitment process is important’* (community midwife)

A desire to contribute to the team was an enabler for both HVs and CMs. However, only HVs mentioned the influence of managers, which could act as both an enabler and a barrier to patient approach.

*‘I do this as it supports my colleagues’* (community midwife)

*‘We share enthusiasm about research and how it impacts on all of us, practitioner and patient alike’* (health visitor)

*‘Some managers encourage participation whereas others are mindful of time restraints’* (health visitor)

1. Goals

Introducing research opportunities to patients was not a high priority for respondents. Both HVs and CMs emphasised that patients’ needs always take precedence, but these respondents did not include a patient’s right to be informed of research opportunities amongst these needs. Commissioned targets took precedence, and since these did not include contributing to research, approaching patients about research opportunities fell to the bottom of the list of activities to be completed during a busy clinical encounter.

*I just about have time to do the job of health visiting. We have targets to meet. A very demanding caseload. Extensive safeguarding. Typing up complex patient notes. Worrying about the lack of resources to actually support the dire needs of my caseload. Sorting out other people’s research is the last thing I need or want to do* (health visitor)

*‘Due to the volume of topics we already have to discuss within a limited time, research would likely slip to the bottom’* (community midwife)

However, 23% of the CMs and 15% of the HVs commented that even in the face of competing clinical targets, they considered approaching patients about research participation to be a priority because practice and care are improved by research, and good research evidence requires participation by their patients. Thus, a belief that research underpins high-quality care meant that the goals domain could also act as a counterbalancing enabler to research recruitment behaviour.

*‘Research into maternity services is a growing area and it is important that all are involved to ensure the service moves forward with robust clinical findings to support out work’* (community midwife)

*‘Despite the time constraints, in order to gather evidence of effective interventions, good practice, etc., we need to be doing research’* (health visitor)

1. Beliefs about capabilities

For the most part, beliefs about capabilities acted as an enabler for both professional groups. Feeling confident to approach patients was mentioned by 55% of CMs and 26% of HVs, and this was the most frequently cited enabler. However, this domain overlapped with the domain of environmental context and resources, and the domain of knowledge. Thus, some respondents expressed a lack of confidence in approaching patients in certain situations, such as more challenging clinical situations or when time was short. Others explained that their confidence in approaching patients about research participation was contingent on their knowledge about the study.

*‘I feel competent and confident and know where to access support’* (community midwife)

*‘I am confident, if I was allocated time and resources’* (health visitor)

*‘I am relatively confident, except in labour’* (community midwife)

*‘I am confident if I know enough to offer a brief explanation or can signpost’* (health visitor)

1. Knowledge

Knowledge could act as a barrier or enabler for both HVs and CMs. A need for good procedural knowledge about the study was mentioned by 11 HVs and 5 CMs, whilst the importance of knowledge of the scientific rationale for the study was emphasised by 9 HVs and 3 CMs. Two HVs and two CMs mentioned needing knowledge of the research topic.

*‘Knowing that participation is voluntary and that participants can with-draw within defined boundaries gives me greater confidence in approaching patients. Understanding the process of ethical approval and how research projects are planned, their protocols laid out etc. gives me greater confidence in explaining participation to others. I would also want to understand the research project objectives and the implication/commitment required from participants to feel confident that I could then ‘sell’ this to a patient’* (health visitor).

1. Other domains

Of the remaining eight TDF domains, only four (Beliefs about consequences; Emotion; Reinforcement; Intentions) were coded for more than 10% of HV participants, and only two (Beliefs about consequences; Reinforcement) were coded by more than 10% of CM participants (Table 3). Nine HVs and seven CMs expressed views that indicated an enabling belief that approaching eligible patients about research participation was an important contribution to research, and hence to improvements in practice. For example, one midwife commented that ‘*Research into maternity services is a growing area and it is important that all are involved to ensure the service moves forward with robust clinical findings to support out work*’ (CM). Counterbalancing the enabling effect of this belief about consequences, nine HVs, but no CMs, expressed concerns about negative consequences for their relationship with patients.

**Table 3.** The frequency (%) of responses from HVs (*n* = 39) and CMs (*n* = 22) coded to each domain of the TDF. The number of codes within each domain that were identified as barriers, enablers or both a barrier and/or enabler is also shown

TDF domain		Frequency (%) per domain								
	HVs (*n* = 39)					CMs (*n* = 22)				
	N (%) quotes	N (%) participants	Barrier codes	Enabler codes	Barrier/enabler	N (%) quotes	N (%) participants	Barrier codes	Enabler codes	Barrier/enabler
Environmental context and resources	50 (19%)	27 (69%)	3	3	0	40 (27%)	18 (82%)	3	5	0
Social/professional role and identity	37 (14%)	23 (59%)	3	5	1	21 (14%)	10 (45%)	3	5	1
Social influences	37 (14%)	22 (56%)	1	3	2	14 (9%)	12 (55%)	1	3	1
Goals	29 (11%)	19 (49%)	2	1	1	16 (11%)	12 (55%)	2	1	
Beliefs about capabilities	29 (11%)	27 (69%)	1	1	2	19 (13%)	15 (68%)	0	1	1
Knowledge	27 (10%)	18 (46%)	0	0	5	11 (7%)	9 (41%)	0	0	4
Beliefs about consequences	19 (7%)	15 (38%)	2	3	0	8 (5%)	5 (23%)	0	1	0
Emotion	14 (5%)	11 (28%)	2	1	0	6 (4%)	2 (9%)	2	0	0
Reinforcement	5 (2%)	5 (13%)	0	3	0	5 (3%)	3 (14%)	0	2	0
Intentions	5 (2%)	5 (13%)	2	1	0	3 (2%)	2 (9%)	0	1	0
Skills	3 (1%)	3 (8%)	1	2	0	2 (1%)	2 (9%)	2	2	0
Optimism	2 (1%)	2 (5%)	1	1	0	2 (1%)	1 (5%)	0	1	0
Memory, Attention and Decision processes	2 (1%)	2 (5%)	0	2	0	1 (1%)	1 (5%)	1	1	0
Behavioural regulation	0 (0%)	0 (0%)	0	0	0	1 (1%)	1 (5%)	0	1	0
Total	259	39	17	26	11	149	22	13	25	7

The influence of the domain Emotion was evident in the responses of a higher proportion of HVs than midwives. (Table 3). Five HVs reported that approaching eligible patients about research participation made them feel stressed, two said it made them feel guilty and four expressed feelings of positivity and enthusiasm when undertaking this activity. One midwife mentioned feeling stressed and one reported feeling apprehensive when approaching eligible patients about research participation. Under the domain Reinforcement, we coded comments from three CMs and five HVs all of which were aimed at enabling the target behaviour. They included being able to offer incentives for staff, monitoring by management and feedback from patients. There was limited evidence for the importance of the domain Intention, with comments from five HVs and two CMs being coded to this domain, whilst the domains Skills, Optimism, Memory attention and decision processes and Behavioural regulation were rarely evident in the dataset for both professional groups.

## Discussion

We have used the TDF (Cane *et al.*, 2012) to identify the factors perceived by HVs and CMs as influencing whether they approach patients about research participation. Key barriers included time and resource constraints, perceived role conflict, conflicting priorities, and particularly for HVs, negative social influences from patients and researchers. Enablers included confidence to approach patients, social influence of peers, managers and researchers and beliefs in the relevance of this behaviour to health care and practice. With this analysis in place, it is possible to use a matrix of behaviour change techniques, which according to expert consensus, link to each of the TDF domains (Michie *et al.*, 2014). Using this approach, we have mapped the key TDF domains to behaviour change techniques to produce recommendations to overcome the modifiable barriers and enhance the enablers (Table 4). These are discussed below alongside the discussion of the key barriers and enablers.

**Table 4.** Recommendations to support HVs and CMs to approach eligible patients about research participation

TDF domain	Barriers to be addressed	Enablers to leverage	Recommendations for future intervention options
Environmental context and resources	Time constraints Insufficient staff Language barriers	High-quality accessible study materials	1. Researchers ensure that study materials and recruitment procedures minimise the study burden for healthcare professionals. Co-production materials and recruitment procedures with relevant staff would support this and potentially contribute a positive social influence.
Social/professional role and identity	Rejection of relevance of this activity to their professional role; concern for role conflict.	Belief in relevance of participant approach to improvements in care and practice	2. Include in study materials and training provided for staff involved in recruitment clear information about the health and care consequences of approaching/not approaching all eligible patients about research opportunities.
Social influences	Beliefs about service users; unsupportive researchers	Team, researchers management influence	3. Seek opportunities to provide positive social influence from peers, management and researchers. For example, researchers involving staff in the design of recruitment procedures and materials, researchers being available, responsive and supportive throughout the recruitment period, management fostering a positive research culture.
Goals	Research support is not a commissioned target	Belief of link to quality of health care	4. Address through recommendation 2.
Beliefs about capabilities	Lack of confidence when time is short, in challenging clinical situations, or if study knowledge is felt to be lacking.	Confidence to approach service users when staff feel supported by researchers and equipped with appropriate knowledge	5. Seek evidence of past successes of approaching eligible patients in more challenging clinical situations and discuss with healthcare practitioners so they are better prepared for this challenge. 6. Address environmental and knowledge related barriers through recommendations 1 and 7.
Knowledge	Poor knowledge of study procedures, study rationale and research topic	Knowledge of study procedures, study rationale and research topic	7. At study set up, researchers provide training that builds procedural knowledge of the recruitment and study processes, and provides a good understanding of the study rationale and the research topic.

The most commonly reported barrier was heavy caseloads and staff shortages, which left insufficient time for HVs and CMs to approach eligible patients about research participation. Time constraints, staff shortages and heavy workloads are widely reported barriers to research recruitment across health specialties and services in the UK, Finland and US (Hoddinott *et al.*, 2007; Sullivan-Bolyai *et al.*, 2007; Nurmi *et al.*, 2015; Skea *et al.*, 2017; Daly *et al.*, 2019). In the present study, respondents clearly communicated the need for healthcare professionals to be allocated sufficient time to deliver this activity, and that in turn demands funding for the staff resource it uses. Whilst there is an established mechanism for the recovery of costs of research in the NHS (Department of Health, 2012), it is important that any salary support funding is visible to the healthcare professionals involved in the patient approach. Counterbalancing the challenge of finding time to approach eligible patients about research opportunities, there was an enabling influence of comprehensive and accessible study information, evident for both HVs and CMs. Previous studies of factors affecting the recruitment activity by midwives have reported that inaccessible study materials present a barrier to recruitment activity by healthcare professions (Halkoaho *et al.*, 2012; Stuart *et al.*, 2015; Daly *et al.*, 2019). By facilitating a good understanding of a study, accessible study materials could support healthcare professionals to approach patients about research by influencing their perception of the time it would take as well as shortening the actual time taken. Our findings suggest that good study materials should be leveraged to ensure that the study burden is minimised.

The professional role and identity of participants was the second most frequently identified domain affecting the patient approach behaviour, with an enabling belief that supporting research is integral to their professional role evident for both CMs and some HVs. However, for some HVs, there were barriers in this domain, including concern about role conflict and, for some, an outright rejection of the relevance of this activity to their professional role. Previous research has found that some clinicians from a range of professional groupings, including midwives, find that navigating the dual role of researcher and healthcare provider can be a challenge (Newington and Metcalfe, 2014; Skea *et al.*, 2017; Daly *et al.*, 2019). There are, however, important developments in policy that could help to address these barriers. In England, this includes the Chief Nursing Officer for England’s (CNO) national strategy for supporting, developing and embedding research (2020–2022) (NHS England, 2020) and the creation of a new nursing, midwifery and care staff research portfolio which showcases the contribution of nursing, midwifery and care staff are making to transforming health and care. By recognising and championing the roles played by nurses and midwives in clinical research, these developments could help to address the issue of role conflict reported here and elsewhere in the literature. It could also be key to delivering the goal set out in the NHS Long-Term Plan (NHS, 2019) to increase patient participation in clinical research in order to facilitate evidence-based policy, improve health outcomes and reduce inequalities.

Our analysis suggests that leveraging an enabling belief in the link between successful research recruitment and improvements in health care could help to address the difficulty of navigating research responsibilities alongside a clinical role. This could be delivered through training at study set up, and reinforced over the course of the study recruitment period by researchers, who in providing timely support would also leverage the enabling effect of their social influence on the behaviour. We suggest that training at study setup also needs to build strong procedural knowledge of the recruitment and study processes, and provide HVs and CMs involved in the study with a good understanding of the study rationale and the research topic. Evidence from systematic reviews of strategies to improve the recruitment activity of clinicians (Fletcher *et al.*, 2012; Newington and Metcalfe, 2014) supports our finding that increasing research knowledge offers a promising route to improved recruitment. However, other systematic review evidence suggests that researcher visits and additional training alone are not sufficient to change the patient approach behaviour of healthcare professionals (Preston *et al.*, 2016; Delaney *et al.*, 2019). This could indicate that the training and support offered by researchers did not meet the needs of healthcare professionals. Indeed, others have found that inadequate support from researchers poses a barrier to research recruitment (Nurmi *et al.*, 2015). Researchers could address this possibility by involving relevant healthcare professionals in the design of the study, the study materials and the training and support for healthcare professionals. Further, the training and support offered by researchers to healthcare professionals should be thoroughly evaluated to ensure it meets the needs of the staff who receive it.

Whilst researcher training and support are clearly important in supporting CMs and HVs to approach patients about research opportunities, there are a number of other barriers that need to be addressed using different measures. From the findings in the present study, a package of interventions would need to target key barriers including time and resource constraints, conflicting priorities, role conflict and negative social influences whilst leveraging enablers including social influence of peers, managers and researchers, training and resources and beliefs in the relevance of this behaviour to health care and practice. Such an approach would provide an opportunity to address our finding that some HVs and CMs choose not to approach all eligible patients about research opportunities, a tendency which is apparently quite widespread amongst other healthcare professionals (Bonevski *et al.*, 2014; Crocker *et al.*, 2015; Hughes-Morley *et al.*, 2015; Stuart *et al.*, 2015; Tromp and Vathorst, 2015). Such selection bias necessarily undermines the representativeness of the study sample, the generalisability of the findings and the scientiﬁc and social value of the study. There is, therefore, a strong case for developing and evaluating a complex intervention that changes this behaviour.

### Strengths and limitations

Data collection for TDF analysis can be done using interviews, focus groups or surveys (Michie *et al.*, 2014; Atkins *et al.*, 2017). Our use of an online survey with open-ended questions, combined the advantages of yielding qualitative data appropriate for an under-researched topic, whilst minimising the burden of the study for the healthcare professionals and the host organisations. Our respondents were generous in their free-text responses, providing ample material to enable us to apply the TDF to analyse the behaviour in question. However, interviews would have yielded richer data and thicker descriptions of the health professionals’ experiences of recruiting participants to research. The anonymity of the online survey may have enabled us to collect a broader range of responses than would have been possible had we conducted interviews. For example, our finding that some respondents chose who to approach about research opportunities based on their perception of the patient’s situation rather the study’s eligibility criteria might not have been volunteered in the presence of the researcher, where demand effects for socially desirable responses would be more keenly felt. Our survey did include some broad open-ended questions to provide an opportunity for respondents to discuss factors that they deemed to be most relevant, an approach recommended by McGowan *et al.* (2020) as supporting an understanding of the behaviour from the participants’ perspectives. However, interviews would have offered the opportunity to probe further the participants’ motivations for selecting particular patients to approach about research opportunities, and to explore views pertinent to the less frequently coded domains, such as skills and emotion.

The lack of a respondent denominator is a limitations as it meant we were unable to calculate an overall response rate and the sample sizes, though adequate for a qualitative study using the TDF (Atkins *et al.*, 2017), are small for samples collected via an online survey. The survey respondents were self-selecting and consequently open to response bias. But the samples were diverse with respect to the range of environments where the respondents were practicing and their experience in their professional role. Further, the gender and ethnicity profile of respondents was in line with the demographics of the NHS workforce in these specialisms. Nevertheless, collecting data about research is from health professionals who are not interested in research is by its nature problematic. Here, the anonymity of the survey may have helped; we received responses from participants with a wide range of views on research, including negative, ambivalent and positive views. Nonetheless, a larger sample size may have further increased the range of views expressed, providing greater insight into the perceptions of the wider population of CMs and HVs.

Although the TDF is widely used in implementation science to understand the behaviour of healthcare professionals, to our knowledge this is the first attempt to use it to understand participant recruitment behaviour. Our use of the TDF enabled us to systematically explore social, environmental, affective and cognitive influences on patient approach by HVs and CMs, and importantly, to explore enablers as well as barriers to his activity.

## Conclusions

This study uses a theory-informed approach to gain new insights into improving research recruitment where HVs and CMs invite patients to take part in a study. We found that inadequate time, poor study materials, research that seems to be irrelevant to their professional role, unsupportive researchers and competing priorities all act to hinder research recruitment activity by CMs and HVs. These barriers could be countered by the enabling effects of confidence, positive social influence from researchers and team members and a belief in relevance of the participant approach to improvements in care and practice. Given the strong evidence for the importance of social and professional factors influencing whether HVs and CMs approach patients about research, we suggest that further work to improve research recruitment could usefully employ a participative approach. The aim would be to develop co-produced interventions tailored to the needs and specific context of each healthcare profession which should then be rigorously evaluated for effectiveness.

## Footnotes

1 We use the term ‘patient’ to refer to the recipients of the health care that community midwives and HVs deliver, rather than the alternative terms ‘client’, ‘consumer’, ‘customer’ and ‘service user’, because a recent scoping review has shown that overall healthcare recipients prefer the term ‘patient’ (Costa *et al.*, 2019).

2 Definition as given by Cane et al (2012).

## Supplementary material

For supplementary material accompanying this paper visit https://doi.org/10.1017/S1463423621000050. click here to view supplementary material
