# Supplementary material for: Community midwives’ and health visitors’ experiences of research recruitment: a qualitative exploration using the Theoretical Domains Framework
Source: Prim Health Care Res Dev. 2021 Jan 29;22:e5. doi: 10.1017/S1463423621000050 (PMC8057511; doi:10.1017/S1463423621000050)
Supplement: Supplementary file 1 [file S1463423621000050sup001.docx]

**Supplementary material 1: Survey questions inviting free text responses to questions about approaching service users about research participation.**

1. Do you think approaching patients/service users about research participation should be part of your role?
2. Considering your other priorities, how important do you think it is for you to approach patients/service users about research participation?
3. How confident are you asking patients/service users about participating in research?
4. Do you intend to approach patients/service users about research participation, when the next opportunity arises? Please can you explain why?
5. How does the thought of approaching a patient/service users about research participation make you feel?
6. What makes it difficult to approach a patient/service users about research participation?
7. What makes it straightforward to approach a patient/service users about a research opportunity?
8. How do you think your patient/service users would feel about being asked to take part in research?
9. How do you think the rest of your team might feel about approaching patient/service users to take part in research?
10. In your opinion, what is the management view on recruiting patients/service users for research?
11. Do you think there should be incentives for professionals to approach service users about research participation, and what might these be?
12. What helps or hinders you to approach patients/service users about research participation?
13. Are there certain situations where you might forget to approach patients/service users about research participation?
14. What might help?
15. Within your team, can you tell us about any ways of working that helps with research recruitment?
16. How do you think recruitment to research should be improved?
17. Do you have any other comments or thoughts about identifying and recruiting participants for research?

**Supplementary material 2: Barriers and enablers to approaching patients about research participation, for health visitors (HVs) and community midwives, mapped to the 14 domains of the Theoretical Domains Framework (TDF).**

| TDF Domain | Barriers | Frequency | | Enablers | Frequency | | Both barrier and enabler | Frequency | |
| --- | --- | --- | --- | --- | --- | --- | --- | --- | --- |
|  |  | HVs | CMs |  | HVs | CMs |  | HVs | CMs |
| Environmental context & resources | Heavy caseloads leaving insufficient time | 19 | 14 | Dedicated research staff | 2 | 3 |  |  |  |
|  | Language | 7 | 4 | Funding to cover the cost | 2 | 1 |  |  |  |
|  | Insufficient staff | 3 | 1 | Comprehensive, accessible study information | 7 | 5 |  |  |  |
|  | Dealing with a challenging clinical situation | 3 | 1 | Physical resources and equipment | 0 | 1 |  |  |  |
| Beliefs about capabilities | I am not confident doing this | 2 | 0 | I feel confident doing this | 10 | 12 | I am confident, in some but not all in situations | 13 | 3 |
|  |  |  |  |  |  |  | I need to believe in the research to feel confident | 2 | 0 |
| Social/professional role and identity | Concerns about conflict of interest | 6 | 1 | Supporting research is integral to my role | 4 | 3 | The topic needs to be relevant to my role | 10 | 2 |
|  | This is the responsibility of researchers | 5 | 1 | If management ask me it is part of my role | 3 | 2 |  |  |  |
|  | Research is not part of my role | 2 | 1 | I will lend limited support | 2 | 3 |  |  |  |
|  |  |  |  | It is a responsibility to our patients | 1 | 1 |  |  |  |
|  |  |  |  | We are well placed to help with recruitment | 0 | 1 |  |  |  |
| Social influences | Client's attitude and needs | 12 | 2 | Contributing to the team | 6 | 8 | Supportive researchers who communicate well | 6 | 4 |
|  |  |  |  | Discussion at team meetings | 3 | 1 | Management support | 2 | 0 |
|  |  |  |  | Research or study champions | 1 | 1 |  |  |  |
| Goals | It’s not my top priority | 14 | 7 | Research is an important goal because it improves health care | 6 | 5 | The value of the research influences whether it is a priority | 2 | 0 |
| Knowledge |  |  |  |  |  |  | Understanding the study | 11 | 5 |
|  |  |  |  |  |  |  | Understanding the rationale | 9 | 3 |
|  |  |  |  |  |  |  | Knowledge of the research topic | 2 | 2 |
| Beliefs about consequences | Negative consequences for relationship with patients | 7 | 0 | Research improves practice | 8 | 5 |  |  |  |
|  | We can bias the sample | 1 | 0 | Research evidences our professional impact | 1 | 0 |  |  |  |
|  |  |  |  | Research is important for my professional development | 1 | 0 |  |  |  |
| Emotion | Adding stress | 5 | 1 | Positive, enthusiastic | 4 | 0 |  |  |  |
|  | Guilt | 2 | 0 |  |  |  |  |  |  |
|  | Apprehension | 0 | 1 |  |  |  |  |  |  |
| Reinforcement |  |  |  | Communicating benefits for families | 0 | 1 |  |  |  |
|  |  |  |  | incentives for clients | 3 | 0 |  |  |  |
|  |  |  |  | Incentives for staff | 0 | 2 |  |  |  |
|  |  |  |  | It is compulsory in my job so it is monitored | 1 | 0 |  |  |  |
|  |  |  |  | Researchers feeding back results of the study | 1 | 0 |  |  |  |
| Intentions | Would prefer not to | 2 | 0 | If asked to help with recruitment I would do so | 2 | 0 |  |  |  |
|  | Would not ask in some situations | 1 | 0 | Would do so given time and incentives | 0 | 2 |  |  |  |
| Skills | Lack of training | 2 | 3 | We have the skills and opportunity | 1 | 2 |  |  |  |
|  | inexperience is a barrier | 0 | 1 | We have the skills to communicate research to our clients | 0 | 1 |  |  |  |
| Optimism | Recruitment is always difficult | 1 | 1 | I am positive about research | 1 | 0 |  |  |  |
| Memory, attention & decision processes | Delays between study set up and recruitment starting cause me to forget | 0 | 1 | Physical prompts and reminders are helpful | 1 | 5 |  |  |  |
|  |  |  |  | Reminders at team meetings | 1 | 0 |  |  |  |
| Behavioural regulation |  |  |  | Increasing familiarity with the study by regularly revisiting aims | 0 | 1 |  |  |  |
